# Hyperthermia induces therapeutic effectiveness and potentiates adjuvant therapy with non-targeted and targeted drugs in an *in vitro* model of human malignant melanoma

**DOI:** 10.1038/s41598-018-29018-0

**Published:** 2018-07-16

**Authors:** T. Mantso, S. Vasileiadis, I. Anestopoulos, G. P. Voulgaridou, E. Lampri, S. Botaitis, E. N. Kontomanolis, C. Simopoulos, G. Goussetis, R. Franco, K. Chlichlia, A. Pappa, M. I. Panayiotidis

**Affiliations:** 1 School of Life Sciences, Heriot Watt University, Edinburgh, Scotland UK; 2 Department of Applied Sciences, Northumbria University, Newcastle Upon Tyne, UK; 3 Department of Obstetrics & Gynecology, Democritus University of Thrace, Alexandroupolis, Greece; 4 Department of Molecular Biology & Genetics, Democritus University of Thrace, Alexandroupolis, Greece; 5 Department of Pathology, University of Ioannina, Ioannina, Greece; 6 Second Department of Surgery, Democritus University of Thrace, Alexandroupolis, Greece; 7 School of Engineering & Physical Sciences, Heriot Watt University, Edinburgh, Scotland UK; 8 Redox Biology Centre, University of Nebraska, Lincoln, USA; 9 School of Veterinary Medicine & Biomedical Sciences, University of Nebraska, Lincoln, USA

## Abstract

In the present study, we have aimed to characterize the intrinsic, extrinsic and ER-mediated apoptotic induction by hyperthermia in an *in vitro* model of human malignant melanoma and furthermore, to evaluate its therapeutic effectiveness in an adjuvant therapeutic setting characterized by combinational treatments with non-targeted (Dacarbazine & Temozolomide) and targeted (Dabrafenib & Vemurafenib) drugs. Overall, our data showed that both low (43 °C) and high (45 °C) hyperthermic exposures were capable of inducing cell death by activating all apoptotic pathways but in a rather distinct manner. More specifically, low hyperthermia induced extrinsic and intrinsic apoptotic pathways both of which activated caspase 6 only as opposed to high hyperthermia which was mediated by the combined effects of caspases 3, 7 and 6. Furthermore, significant involvement of the ER was evident (under both hyperthermic conditions) suggesting its role in regulating apoptosis via activation of CHOP. Our data revealed that while low hyperthermia activated IRE-1 and ATF6 only, high hyperthermia induced activation of PERK as well suggesting that ultimately these ER stress sensors can lead to the induction of CHOP via different pathways of transmitted signals. Finally, combinational treatment protocols revealed an effect of hyperthermia in potentiating the therapeutic effectiveness of non-targeted as well as targeted drugs utilized in the clinical setting. Overall, our findings support evidence into hyperthermia’s therapeutic potential in treating human malignant melanoma by elucidating the underlying mechanisms of its complex apoptotic induction.

## Introduction

Malignant melanoma is known to be the most aggressive form of skin cancer and one of the most lethal solid tumor types with its incidence rates increasing globally over the past few decades rendering the disease the 5^th^ most common type of cancer in the UK^1^. Hyperthermia is defined as the application of an exogenous heat source which acts by directly killing tumor cells or enhancing the efficacy of other therapeutic means (e.g. radiation, chemotherapy, etc.) against various cancer types^2,3^. The latest technological advances have allowed the more accurate and efficient application of hyperthermia in the tumor site as well as the precise temperature monitoring all of which have resulted in promising clinical outcomes in a wide range of cancer types^4^.

Results from numerous *in vitro* and *in vivo* studies have identified apoptosis as the key underlined pathway responsible for the induction of cell death as a response to hyperthermic treatments^5–7^. In general, apoptosis involves the induction of the extrinsic and intrinsic pathways whose activation depends on distinct signals^8^. Evidence, by other groups, has implicated the activation of both apoptotic pathways (in response to hyperthermia) the extent of which is dependent on the cancer type, temperature and duration of exposure^9^. In addition, the activation of an ER-mediated non-conventional apoptotic pathway has been documented in a study utilizing melanoma and non-melanoma cell lines^10^. Finally, although many studies have demonstrated the involvement of apoptosis in hyperthermia-induced cell death (in various cancer types) there is limited data pertaining to the elucidation of its underlined mechanism(s) in human malignant melanoma. Thus, the aim of this study was to delineate the underlined mechanism(s) of hyperthermia’s effectiveness in inducing apoptosis, and furthermore to potentiate the action of clinically relevant non-targeted and targeted drugs in an *in vitro* model of human malignant melanoma. Consequently, our objectives were to (i) develop an optimized experimental platform of hyperthermic exposures by utilizing a validated model of human malignant melanoma, (ii) determine the mode of apoptotic induction and the role of the ER-stress response in relation to the duration and intensity of the hyperthermic exposures and (iii) evaluate the role of hyperthermia in potentiating the therapeutic efficacy of clinically-relevant non-targeted and targeted drugs. The latter is of paramount importance given that the disease is a highly aggressive and metastatic type of skin cancer which despite recent improvements in treatment options remains an incurable disease with a poor prognosis and an unmet need for more efficient treatments.

## Results

### Development of an experimental hyperthermic platform

**Figure 1:**
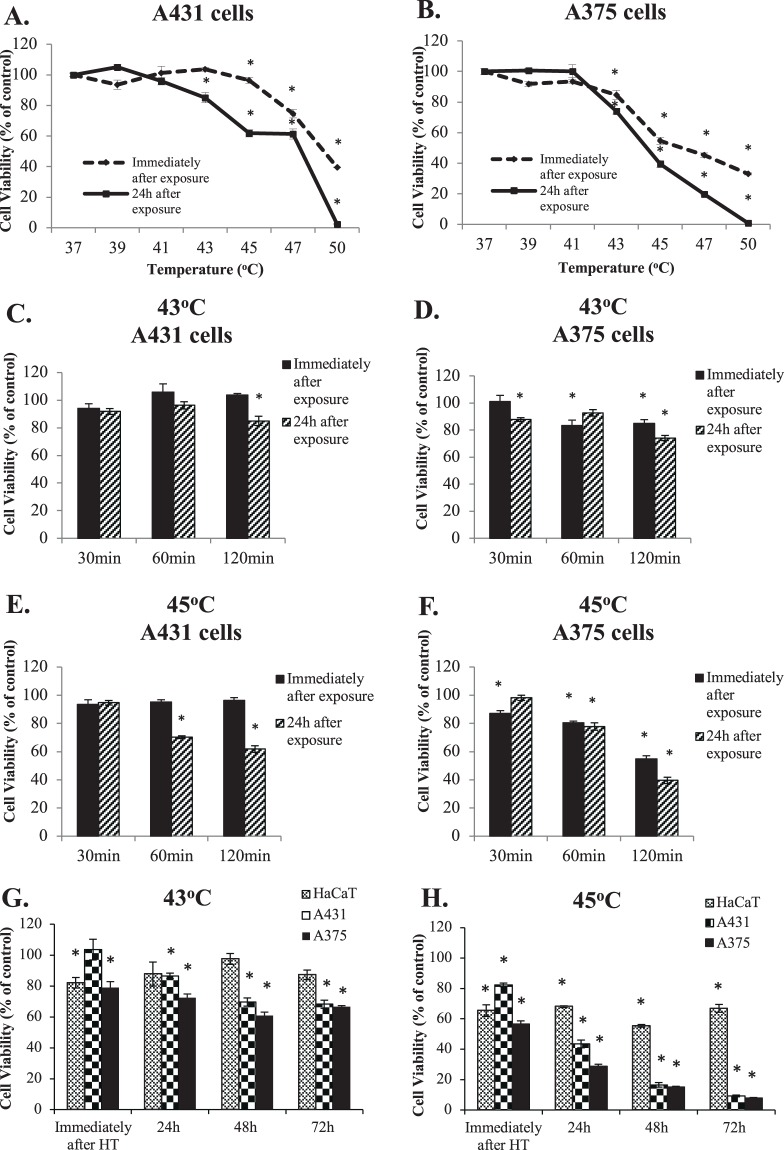
Kinetics of hyperthermia in human immortalized keratinocyte (HaCaT), epidermoid carcinoma (A431) and malignant melanoma (A375) cell lines. The effect of hyperthermia on cell viability levels in (**A**) A431 and (**B**) A375 cell lines; The effect of hyperthermia at different time courses in (**C** and **E**) A431 and (**D** and **F**) A375 cell lines; (**G** and **H**) HaCaT, A431 and A375 cell lines were subjected to hyperthermia and cell viability levels were determined immediately after as well as 24–72 h post-exposure. Data shown are mean values (n = 5) ± SEM and represent one of three independent experiments. Asterisk (*) indicates statistical significance at p < 0.05.

In this set of experiments, we determined the optimal conditions of hyperthermic exposures by utilizing the human malignant melanoma (A375) and epidermoid carcinoma (A431) cell lines. Several temperature-response and time-course experiments were performed with cell viability levels assayed immediately after the 2 h hyperthermic exposure as well as after 24 h post-exposure, at 37 °C (Fig. 1A,B). Data showed that exposing cells to temperatures lower than 43 °C did not induce a significant effect on viability levels in both cell lines. However, when cells were exposed to temperatures higher than 43 °C, there was a significant reduction in viability observed at a greater extent in A375 cells only. Furthermore, a significant decline in viability was recorded, in both cell lines, at temperatures above 45 °C suggesting excessive cellular destruction (Fig. 1A,B). To these ends, when cells were exposed at 43 °C over shorter time courses (30–60 min) there was no significant reduction in viability levels (Fig. 1C,D) whereas exposure of both cell lines at 45 °C caused a considerable decline in the numbers of living cells (Fig. 1E,F). More specifically, our data showed that there was a 15% and 25% reduction in cell viability 24 h post-exposure to 43 °C (Fig. 1C,D) and further reduced to 60% and 40% at 45 °C (Fig. 1E,F) in A431 and A375 cells respectively.

In another set of experiments, cells were exposed to either 43 °C or 45 °C over 2 h and cell viability was determined following 24–72 h post-incubation at 37 °C in order to determine any further and more prolonged decrease in cell viability. A non-malignant immortalized keratinocyte (HaCaT) cell line was included in an attempt to determine the safety profile of the hyperthermic exposures on the rationale that keratinocytes are the cells surrounding melanocytes and so were used as a control group. Results confirmed our previous observations in that A375 cells were more sensitive to 43 °C (as there was a 30–40% decline in cell viability levels at 24–72 h post-exposure) while A431 cells were more resistant (Fig. 1G). Moreover, exposure at 45 °C induced an even more profound decrease (70–90%) in the viability of A375 cells. In agreement with our previous observations, A431 cells remained more resistant at 24 h post-exposure but this effect was not seen at 48–72 h suggesting that at these time points the hyperthermic effect was equally cytotoxic in both cell lines (Fig. 1H). On the contrary, HaCaT cells were significantly more resistant to exposure with either 43 °C (Fig. 1G) or 45 °C (Fig. 1H), irrespectively of the experimental condition, suggesting that these cells can retain their tolerance to increased temperatures as opposed to A375 and A431 cells.

**Figure 2:**
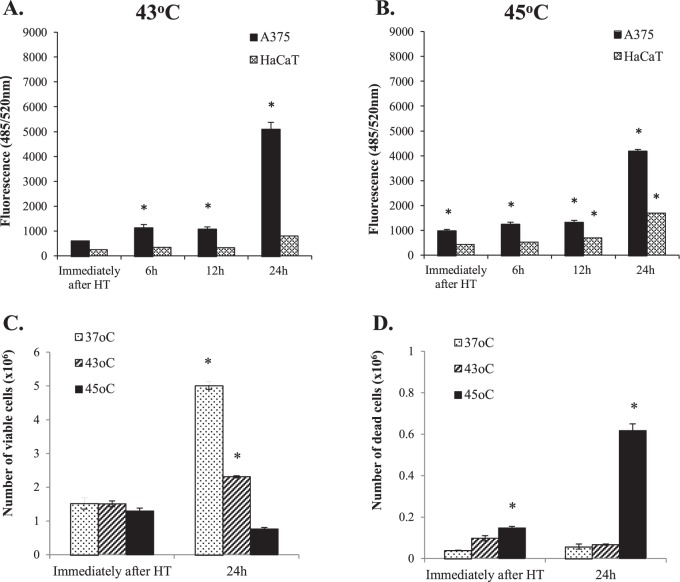
Hyperthermia-induced cytotoxicity in human immortalized keratinocyte (HaCaT) and malignant melanoma (A375) cell lines. The effect of hyperthermia at (**A***)* 43 °C and *(***B***)* 45 °C was expressed as fluorescence values indicative of relative levels of dead cells in A375 and HaCaT cells; The effect of hyperthermia on levels of *(***C***)* cell viability and *(***D***)* dead cells was determined by trypan blue staining in A375 cells. Data shown are mean values (n = 5) ± SEM and represent one of three independent experiments. Asterisk (*) indicates statistical significance at p < 0.05.

To examine further the impact of hyperthermia in triggering cytotoxicity, relative levels of dead cells were determined by utilizing the CytoTox Fluor assay and trypan blue staining protocols. According to our findings, there was a significant increase in cytotoxicity levels in A375 compared to the HaCaT cells when exposed at both 43 °C (Fig. 2A) and 45 °C (Fig. 2B) either immediately after exposure or 6–24 h post-exposure. In addition, when utilizing a trypan-blue staining method, data revealed that A375 cells exposed to 43 °C showed reduced proliferating potential compared to 37 °C (at 24 h post-exposure) while there was no significant change in the levels of cytotoxicity (dead cells) (Fig. 2C,D). However, exposure at 45 °C was associated with a slight increase in the levels of dead cells immediately after exposure an effect which became more apparent at 24 h post-exposure (Fig. 2D).

### Hyperthermia induces apoptosis in human malignant melanoma (A375) cells

**Figure 3:**
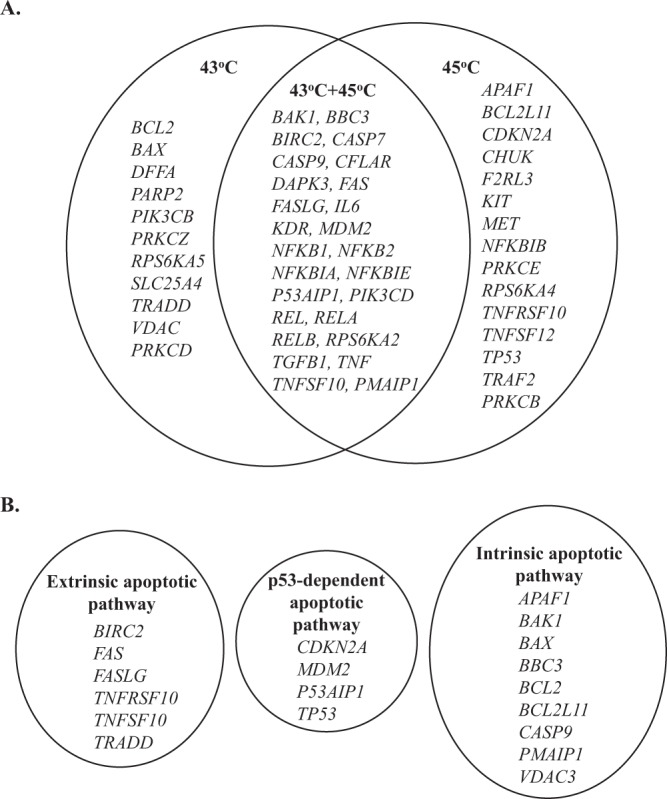
Apoptotic gene profiling by RT-PCR-based microarrays. A list of apoptotic genes categorized according to (**A**) hyperthermic exposure condition and (**B**) key apoptotic pathway involvement.

In an attempt to investigate the effect of hyperthermia in inducing changes in the expression of key apoptotic genes, we utilized a genomic approach based on a real-time PCR microarray gene expression profiling system. Our data showed that there were several differences in the induction of various apoptotic genes 24 h post-exposure to 43 °C and 45 °C. A number of intrinsic apoptotic genes (Fig. 3B) were found to be either up- (e.g. *APAF1*, *BAK1*, *BAX*, *BBC3*, *BCL2L11*, *CASP9*, *PMAIP1*) or down-regulating (e.g. *BCL2*, *VDAC3*) (Table 1). Of these, only *BAK1*, *BBC3*, *CASP9* and *PMAIP1* were common between the two hyperthermic temperatures with *BAX*, *BCL2*, *VDAC* and *APAF1*, *BCL2L11* being exclusively involved at 43 °C and 45 °C respectively (Fig. 3A). On the other hand, a number of extrinsic apoptotic genes (Fig. 3B) were all shown to be up-regulated (e.g. *FAS*, *FASLG*, *BIRC2*, *TNFRSF10*, *TNFSF10* and *TRADD*) (Table 1). However, their up-regulation was either common between the two hyperthermic temperatures (e.g. *FAS*, *FASLG*, *BIRC2*, *TNFSF10*) or restricted to either 43 °C (e.g. *TRADD*) or 45 °C (e.g. *TNFRSF10*) (Fig. 3A). Finally, a number of genes was shown to be involved in the p53-dependent apoptotic response (e.g. *CDKN2A*, *MDM2*, *P53 AIP1*, *TP53*) (Fig. 3B) with some of which being either up-regulated (e.g. *CDKN2A*) or down-regulated (e.g. *TP53*) at 45 °C (Table 1) while the expression of *MDM2* and *P53AIP1* was common between the two hyperthermic temperatures (Fig. 3A).

**Table 1:** Expression levels of apoptotic genes in A375 cells at 24 h post-exposure to 43 °C and 45 °C hyperthermia.

Gene	Hyperthermia at 43 °C	Hyperthermia at 45 °C	Fold difference
*APAF1*	—	2.9	↑2.9
*BAK1*	2.1	32.0	↑15.2
*BAX*	2.4	—	↑2.4
*BBC3*	4.3	16.0	↑3.7
*BCL2*	0.5	—	↓2.0
*BCL2L11*	—	2.0	↑2.0
*BIRC2*	1.7	2.0	—
*CASP7*	1.5	2.0	↑1.3
*CASP9*	1.5	1.5	—
*CDKN2A*	—	2.9	↑2.9
*CFLAR*	1.5	1.5	—
*CHUK*	—	2.0	↑2.0
*DAPK3*	2.2	2.0	—
*DFFA*	1.5	—	↑1.5
*F2RL3*	—	2.1	↑2.1
*FAS*	3.0	11.6	↑3.9
*FASLG*	10.4	176.3	↑16.9
*IL6*	23.7	23.0	—
*KDR*	3.0	0.5	↑6.0
*KIT*	—	2.1	↑2.1
*MDM2*	6.1	16.2	↑2.7
*MET*	—	1.5	↑1.5
*NFKB1*	1.5	1.5	—
*NFKB2*	1.5	2.0	↑1.3
*NFKBIA*	2.2	6.3	↑2.9
*NFKBIB*	—	2.0	↑2.0
*NFKBIE*	2.1	2.0	—
*P53AIP1*	2.2	5.8	↑2.6
*PARP2*	0.5	—	↓2.0
*PIK3CB*	1.5	—	1.5
*PIK3CD*	2.2	2.0	—
*PMAIP1*	1.7	3.8	↑2.2
*PRKCB*	—	12.3	↑12.3
*PRKCD*	1.5	—	↑1.5
*PRKCE*	—	1.5	↑1.5
*PRKCZ*	1.5	—	↑1.5
*REL*	1.5	1.5	—
*RELA*	1.5	1.5	—
*RELB*	1.7	6.3	↑3.7
*RPS6KA2*	0.5	0.4	—
*RPS6KA4*	—	1.5	↑1.5
*RPS6KA5*	0.5	—	↓2.0
*SLC25A4*	1.5	—	↑1.5
*TGFB1*	1.5	1.5	—
*TNF*	1.5	186.1	↑124.0
*TNFRSF10*	—	2.0	↑2.0
*TNFSF10*	1.6	4.1	↑2.6
*TNFSF12*	—	2.0	↑2.0
*TP53*	—	0.5	↓2.0
*TRADD*	1.5	—	↑1.5
*TRAF2*	—	2.0	↑2.0
*VDAC3*	0.5	—	↓2.0

Data, from each hyperthermic condition (43 °C or 45 °C), are expressed as fold increase in comparison to control (37 °C) (1^st^ and 2^nd^ column) while expressed as fold difference when comparing the two hyperthermic exposure conditions (43 °C and 45 °C) with each other (3^rd^ column). Gene expression data were analyzed by the ΔΔCt method and differences observed were expressed as fold change in gene expression by using the DataAssist v3.01 software. (↑) denotes up-regulation whereas (↓) down-regulation and (−) no significant fold change between hyperthermic conditions (43 °C or 45°C) compared to control (37°C). Data shown are mean values from two independent experiments.

**Figure 4:**
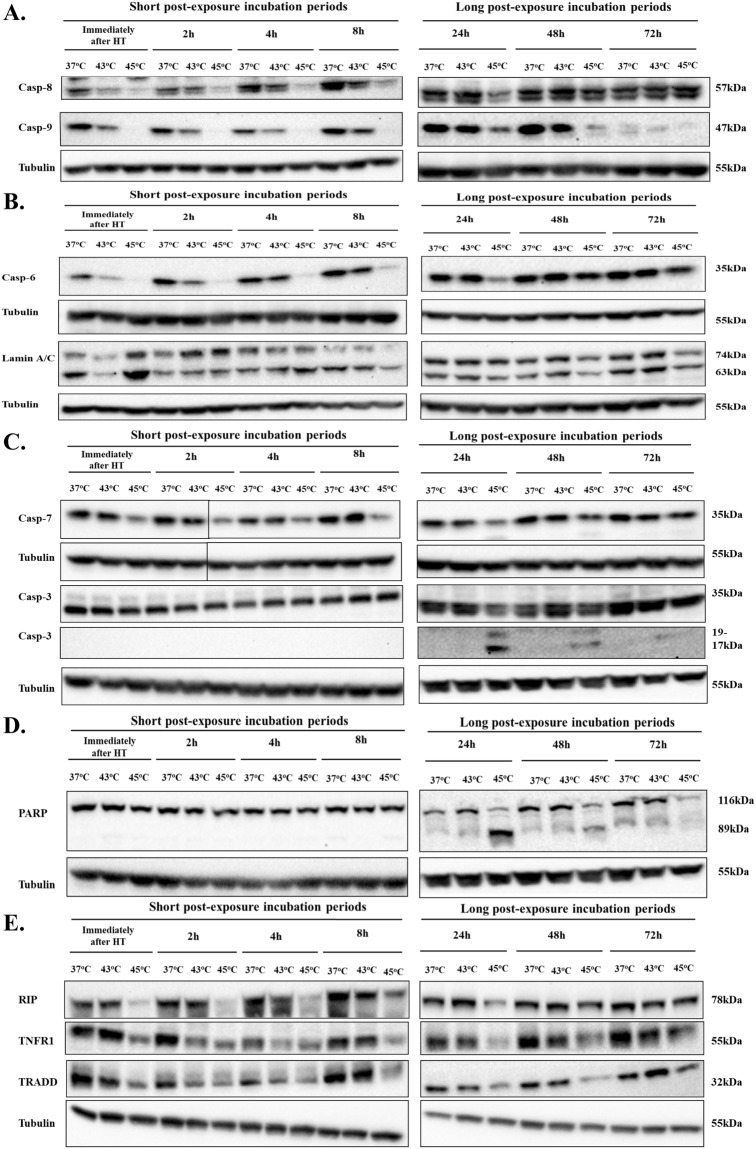
Hyperthermia-induced apoptosis in a human malignant melanoma (A375) cell line. The effect of hyperthermia on protein content of (**A**) caspases-8 and -9; (**B**) caspase-6 and lamin A/C; (**C**) caspases-7 and -3; (**D**) uncleaved/cleaved PARP; and (**E**) RIP, TNFR1, TRADD. Cells were grown overnight at 37 °C followed by exposure to hyperthermia, for 2 h, and then transferred back to 37 °C for the indicated post-exposure incubation times (2–72 h). Cell lysates were prepared and subjected to western blotting. Control cells were kept at 37 °C. β-tubulin was used as loading control. Samples from short and long-post exposure incubation periods following hyperthermia were electrophorized on separate gels. Delineation shows blots cropped from different areas of the same blot or different blots. Full-length blots are provided in Supplementary Material (Fig. 1S & 2S). Data shown is representative of at least two independent experiments.

Furthermore, we profiled the response of various caspases by utilizing western blotting assays. More specifically, initiator caspases-8 and -9 showed identical patterns of expression whereby they were activated immediately after as well as up to 8 h post-exposure at both hyperthermic temperatures. At longer post-exposure incubation periods (24–72 h), they were not shown to be activated except at 45 °C when they remained active even at 24 h (Fig. 4A). Moreover, we tested the activation of executioner caspase-6 by determining its protein levels as well as those of its target protein, lamin A/C. Data demonstrated a significant reduction in its protein expression levels at 43 °C (up to 4 h post-exposure) whereas remained consistently active up to 72 h post-exposure, at 45 °C (Fig. 4B). The same pattern was observed when the uncleaved form of lamin A/C was assayed confirming the results obtained with caspase-6 (Fig. 4B). In the case of the executioner caspase-7, it was also found to be consistently activated immediately after exposure to 45 °C as well as after 2–72 h post-exposure without any significant activation observed at 43 °C (Fig. 4C). Data also revealed that in the case of the executioner caspase-3, its cleaved and un-cleaved protein expression levels were neither changed immediately after hyperthermic exposures nor at any time point up to 24 post-exposure. However, at this time point onwards its cleaved form became evident, only at 45 °C, suggesting of its activation at this hyperthermic condition (Fig. 4C). In agreement to these observations, poly ADP ribose polymerase (PARP) was also shown to remain unaffected up to 24 h post-exposure to 43 °C while it remained cleaved at every other time point of post-exposure to 45 °C (Fig. 4D).

In an attempt to characterize, in more detail, the involvement of the death receptor apoptotic pathway in response to hyperthermia, we examined changes in protein expression levels of three different death receptor molecules. According to our results, TNFR1 and TRADD presented a similar pattern of expression whereby there was a reduction in their protein content up to 24 h post-exposure to 43 °C while this decrease was further sustained up to 72 h post-exposure to 45 °C (Fig. 4E). In the case of RIP, there was a profound decline only at 45 °C at each time point up to 48 h post-exposure (Fig. 4E).

### Hyperthermia induces ER stress response in human malignant melanoma (A375) cells

**Figure 5:**
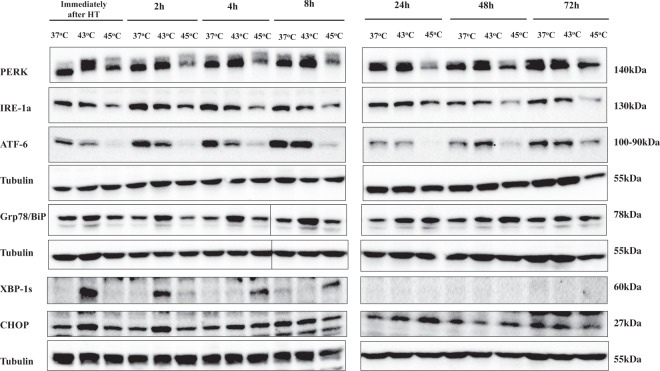
Hyperthermia-induced activation of ER stress response pathwaws in human malignant melanoma (A375) cells. The effect of hyperthermia on protein content of PERK, IRE-1a, ATF-6, GRP78/BiP, XBP-1S and CHOP. Cells were grown overnight at 37 °C followed by exposure to hyperthermia, for 2 h, and then transferred back to 37 °C for the indicated post-exposure incubation times (2–72 h). Cell lysates were prepared and subjected to western blotting. Control cells were kept at 37 °C. β-tubulin was used as loading control. Samples from short and long-post exposure incubation periods following hyperthermia were electrophorized on separate gels. Delineation shows blots cropped from different areas of the same blot or different blots. Full-length blots are provided in Supplementary Material (Fig. 3S). Data shown is representative of at least two independent experiments.

Alterations in protein expression levels of several regulators taking part in ER stress induction (UPR response) were studied. First, we examined changes in protein content of Grp78/BiP, a chaperone protein induced by irregular protein folding and also known to bind to stress response proteins like PERK, IRE-1a and ATF-6 under normal conditions. However, upon ER stress induction it dissociates and activates their respective UPR pathways. According to our results, there was a significant increase in Grp78 protein expression levels up until 8 h post exposure to 43 °C and 24–48 h post exposure to 45 °C (Fig. 5). Furthermore, data showed a reduction in PERK protein levels up to 24 h post exposure at 45 °C whereas there were no alterations in its protein content at any time point post exposure to 43 °C (Fig. 5). Moreover, our findings demonstrated that IRE-1a and ATF-6 followed a similar pattern of expression characterized by a decrease in protein content up to 8 h post exposure to both hyperthermic temperatures with such decline being maintained at longer post exposure incubation periods (24–48 h) but only in the case of 45 °C (Fig. 5).

We also examined the protein expression of XBP-1s (the downstream target of IRE-1a) which was found to be induced immediately after exposure as well as 2 h and 4–8 h post exposure to 43 °C and 45 °C respectively. On the contrary, its protein levels were completely undetected at any time point after 8 h post exposure to both hyperthermic conditions (Fig. 5). Finally, our data revealed a significant alteration in the protein expression levels of CHOP (a major regulator of the ER-stress response), immediately after and 2–8 h post exposure to 43 °C whereas its induction became evident only after 24 h post exposure to 45 °C (Fig. 5).

### Hyperthermia activates the heat shock response in human malignant melanoma (A375) cells

**Figure 6:**
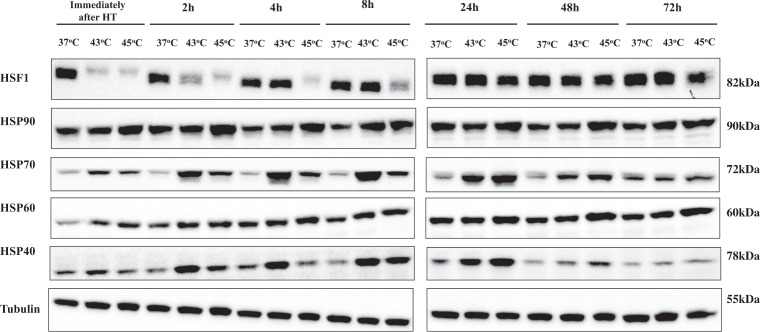
Hyperthermia-induced regulation of heat shock proteins in human malignant melanoma (A375) cells. The effect of hyperthermia on protein content of HSF1, HSPs 90, 70, 60 and 40. Cells were grown overnight at 37 °C followed by exposure to hyperthermia, for 2 h, and then transferred back to 37 °C for the indicated post-exposure incubation times (2–72 h). Cell lysates were prepared and subjected to western blotting. Control cells were kept at 37 °C. β-tubulin was used as loading control. Samples from short and long-post exposure incubation periods following hyperthermia were electrophorized on separate gels. Delineation shows blots cropped from different areas of the same blot or different blots. Data shown is representative of at least two independent experiments.

In an attempt to monitor the effect of hyperthermia on heat shock response, we determined alterations in the expression of various protein regulators. Overall, there was a reduction in the protein content of transcription factor HSF1 immediately after and up to 4 h post exposure to 43 °C while this trend continued thereafter (2–72 h) but only at 45 °C (Fig. 6). In contrast, the expression levels of HSP 90 increased 4–48 h post exposure to 45 °C whereas remained at control levels at 43 °C (Fig. 6). Furthermore, HSPs 40 and 70 exhibited a similar pattern of expression in a manner where their protein contents were elevated immediately after and up to 24–48 h at both hyperthermic conditions (Fig. 6). Finally, the expression of HSP 60 was elevated 2–24 h post exposure to both hyperthermic temperatures and 24–72 h post exposure to 45 °C only (Fig. 6).

### Hyperthermia potentiates the effectiveness of non-targeted and targeted therapeutic drugs in human malignant melanoma (A375) cells

**Figure 7:**
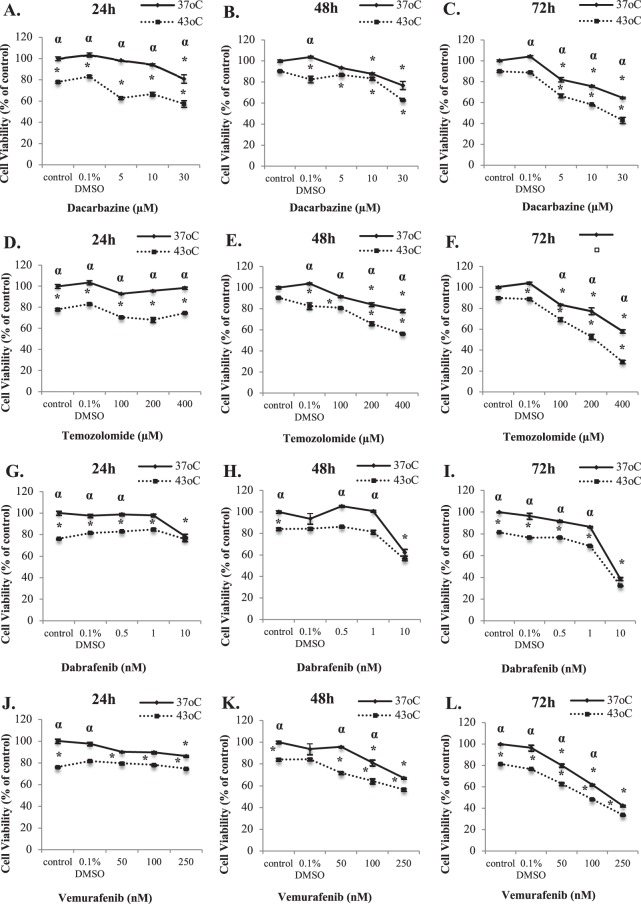
Hyperthermia-induced potentiation of therapeutic effectiveness of non-targeted and targeted drugs in malignant melanoma (A375) cells. Comparison of cell viability levels following treatment with Dacarbazine (**A**–**C**), Temozolomide (**D**–**F**), Dabrafenib (**G**–**I**) and Vemurafenib (**J**–**L**). The drug compounds were used either as single agents, at 37 °C, or in combination with 43 °C hyperthermia over a time-course of 24–72 h. Cell viability levels were calculated by comparison with 0.1% DMSO (vehicle) at 37 °C. Data represent mean values ± SEM (n = 5) and represent one of at least two independent experiments. Asterisk (*) indicates statistical significance at p < 0.05 for comparison with respective control at 37 °C or 0.1% DMSO at 37 °C. Alpha (α) indicates statistical significance at p < 0.05 for comparisons between 37 °C and 43 °C for each experimental condition.

In order to investigate if hyperthermia potentiates the therapeutic effectiveness of drugs currently used in the clinical setting, we utilized two chemotherapeutic agents (Dacarbazine and Temozolomide; non-targeted agents) and two inhibitors of B-Raf^V600E^ (Dabrafenib and Vemurafenib; targeted agents) in combinational treatment protocols along with hyperthermia at 43 °C. Results showed that exposing cells to either Dacarbazine alone or in combination with hyperthermia had a significant additive effect on reducing cell viability at 48–72 h post-exposure, while at 24 h there appeared to be no significant changes with any of the treatment protocols (Fig. 7A–C). Moreover, it appeared that the effect of Dacarbazine on cell viability was potentiated in the presence of hyperthermia at 48–72 h post-exposure (Fig. 7A–C). In addition, Temozolomide (either alone or in combination with hyperthermia) also significantly reduced cell viability at 48–72 h post-exposure in a manner similar to Dacarbazine. However, the observed hyperthermia-induced potentiation was more apparent than in the case of Dacarbazine (Fig. 7D–F).

On the other hand, similar observations were made in the case of targeted B-Raf^V600E^ inhibitors namely Dabrafenib and Vemurafenib. In particular, the efficacy of Dabrafenib was remarkably enhanced when administered in combination with hyperthermia at 24–48 h post-exposure (Fig. 7G–I) although the utilization of each therapeutic protocol alone (i.e. drug at 37 °C and 43 °C) did not induce a consistent pattern of reduced cell viability in accordance with the range of concentrations tested at each one of the indicated post-exposure time points (Fig. 7G–I). In addition, when hyperthermia was combined with Vemurafenib treatment there was also an observed potentiation in reducing cell viability levels at 24–72 h post-exposure (Fig. 7J–L). Finally, it is noteworthy that although a similar pattern of potentiation was observed between the two targeted drug agents it occurred at substantially different concentration ranges in a manner where those of Vemurafenib were 100-fold higher that the corresponding Dabrafenib ones. Collectively, our data indicate a potential role of hyperthermia in enhancing the therapeutic effectiveness of non-targeted and targeted therapeutic drugs used in the clinical setting in the context of disease management.

## Discussion

Data from various clinical studies have shown that hyperthermia enhances the effectiveness of therapeutic strategies like radiation and chemotherapy^11–14^. In the case of malignant melanoma, there is only a limited number of reports investigating into the induction of cell death as a response to hyperthermia^10,15,16^.

In optimizing our hyperthermic exposure platform, detailed kinetic analyses were performed by utilizing the epidermoid carcinoma (A431) and malignant melanoma (A375) cell lines. In addition, we have included a non-tumorigenic immortalized keratinocyte (HaCaT) cell line in the context of providing a safety profile for hyperthermic exposures given that keratinocytes are the primary epidermal cells surrounding melanocytes^17^. To our knowledge, there are no previous studies evaluating the effect of hyperthermia-induced cytotoxicity in non-malignant cell lines. Finally, the observed reduction in cell viability, at 43 °C, could also be attributed to hyperthermia’s capacity to induce cell cycle growth arrest. In fact, several studies have associated hyperthermia’s anti-proliferative effects with alterations in cell cycle regulation in various cell lines^18–20^.

Hyperthermia-induced cell death has been the subject of many studies utilizing a wide range of experimental cancer models^7,21,22^. Our results indicate the triggering of the extrinsic and intrinsic apoptotic pathways supported by the activation of caspases 8, 9, TNF-R1 and TRADD (at both 43 °C and 45 °C) suggesting their interaction in forming a death domain capable of recruiting caspase-8. Although our findings are in agreement with other studies demonstrating the induction of death receptors as a response to thermal stress^23–26^, they have not been documented in an experimental model of malignant melanoma before. Moreover, our data showed activation of RIP1, at 45 °C, which could be indicative either of the protein’s interaction with FADD and TRADD in stimulating the extrinsic pathway or its interaction with RIP3 for the formation of the necrosome required for necroptotic cell death^27,28^. On the other hand, induction of caspase-9 has been associated with activation of the intrinsic apoptotic pathway in Jurkat cells^29^ and various other cancer cell lines^30^ while a recent study (utilizing melanoma cells) has provided no evidence for the activation of either caspase-8 or -9 under heat stress^10^. Such conflicting data can be attributed to the utilization of different experimental conditions (e.g. variations in hyperthermic experimental platforms, exposure kinetics and utilization of different types of cells^3^) indicating the significance of utilizing an optimized experimental platform when assessing the effect of *in vitro* hyperthermic exposures. Finally, we observed that only caspase-6 became activated at 43 °C whereas caspases-3, -7 and -6 were all induced at 45 °C. Although our results are consistent with previous reports, demonstrating the induction of caspases-3 and -7 in response to hyperthermia^10,25^, the activation of caspase-6 (at 43 °C only) has not been previously reported.

Moreover, we investigated the participation of the ER stress response pathway in triggering hyperthermia-induced cell death. Our data showed an increase in Grp78 indicative of an increased demand for chaperone proteins together with a slight decrease in PERK which may be caused by its increased homodimerization for phosphorylating the eIF2 factor thus inhibiting protein synthesis in stressed cells^26^. Similarly, induction of IRE-1a and ATF-6 was also noted suggesting that IRE-1a becomes homodimerized and binds to downstream proteins while ATF-6 is cleaved to its active form under ER-stress conditions. Consistent with these observations, XBP-1s (the downstream target of IRE-1a) was shown to be up-regulated and together with active ATF-6 can modulate the activation of UPR pathways^31,32^. Finally, induction of CHOP was shown to be dependent on the activation of ATF-6 and XBP-1s and potentially linked to stimulation of apoptosis^33,34^. Interestingly, the induction of IRE-1a and ATF-6 has been suggested to play an anti-apoptotic role under ER stress conditions, in contrast to PERK which was shown to have pro-apoptotic effects instead^35–39^. In parallel, we also examined alterations in several heat shock proteins (HSPs) as a response to stress-induced protein misfolding and aggregation both of which can induce cell death. In particular, the up-regulation of HSPs 70 and 90 has been previously demonstrated to exert anti-apoptotic effects by preventing the formation of the apoptosome^40,41^. In addition, inhibition of HSP 70 appears to have anti-cancer effects by preventing tumor growth and enhancing cisplatin’s cytotoxicity in an *in vivo* model of melanoma^42^. Findings from a recent study have linked the absence of JB12 (an ER-associated HSP 40 protein) with the stimulation of ER-stress-mediated apoptosis^43^ whereas HSP 60 exerts its anti-apoptotic effects by acting as a mitochondrial chaperone while its inhibition promotes apoptosis and prevents tumor growth in an *in vivo* glioblastoma model^44,45^. Interestingly, the suppression of HSF1 appears to exert anti-proliferative effects in melanoma cells under hyperthermic conditions^46^. To this end, both HSPs 90 and 70 can interact with HSF1 and suppress its function^47,48^.

On a different note, we aimed to investigate the effect of hyperthermia in potentiating the effectiveness of several drugs (currently utilized in the clinical setting), in a way where lower concentrations can exert comparable cytotoxicity (with that observed at higher concentrations) and thus potentially minimizing the risk for unwanted side effects^49^. According to our initial observations, we determined that 43 °C was the optimal hyperthermic temperature used in all adjuvant treatment protocols (data not shown). This finding is in agreement with other studies indicating that the combination of low hyperthermia (40–43 °C) with chemotherapy exerts increased cytotoxicity against various cancer cells^3,50^ while higher temperatures (>45 °C) are associated with the induction of necroptotic death^10,51^. Our data revealed that hyperthermia potentiated the effectiveness of DTIC, the action of which requires its obligatory bio-activation in the liver^52^. This is an experimental limitation of our *in vitro* model and consequently the reason for utilizing TMZ in additional experiments. This drug agent is an analogue of DTIC but without the requirement for bio-activation as it is spontaneously metabolized to its active form^52^. TMZ’s efficacy was also demonstrated to be potentiated in the presence of hyperthermia to a higher degree than DTIC. This observation is also in agreement with previous studies demonstrating hyperthermia-induced enhancement of the therapeutic efficacy of TMZ in *in vitro* and *in vivo* experimental models^53^. On another note, almost half of melanoma patients carry a mutation (V600E) in the *BRAF* oncogene which results in an amino acid substitution, at amino acid 600, from a valine (V) to a glutamic acid (E). Consequently, there has been a growing interest in developing new drugs capable of targeting this mutation and thus inhibiting the continuous activation of MAPK/ERK signaling pathway which contributes to tumor growth^54^. Two such *BRAF*-targeted drugs are Vemurafenib and Dabrafenib both of which have been approved by FDA in 2011 and 2013 respectively^55,56^. Our data revealed that exposure to mild hyperthermia (43 °C) potentiated the therapeutic effectiveness of both drugs, a finding which has not been reported before.

Moreover, hyperthermia has been shown to induce oxidative stress via generation of reactive oxygen species (ROS)^57^ which, in turn, can induce an apoptotic response^58^. For instance, a previous study utilizing *in vitro* and *in vivo* models of malignant melanoma has demonstrated that exposure to 45 °C was capable of affecting the redox state but not altering the cellular proliferating potential^59^. In addition, generation of free radicals along with the presence of molecular oxygen appeared to affect the efficiency of several photosensitizers against melanoma cells^60^. Furthermore, the combination of hyperthermia with radiation therapy was found to be more effective due to the suppressed oxygen uptake caused by the increased temperature in multicell spheroids^61^. On another note, under normal conditions, melanocytes produce melanin that is capable of protecting cells by absorbing UV radiation^62^. L-tyrosine acts as a positive regulator of melanogenesis while it is also associated with increased metastatic potential of melanoma cells^63^. Numerous reports have shown the utilization of various forms of melanin-containing nanoparticles based on their ability to increase the temperature on tumor location (due to the capacity of melanin to absorb energy after irradiation) thus leading to tumor growth inhibition and even complete eradication^64–69^. On the other hand, various studies have shown that hyperthermia can influence the immune system in various ways including induction of HSPs, improvement of dendritic cell and NK-cell function, improved lymphocyte-endothelial adhesion and leukocyte trafficking, and mediation of immune surveillance^70^. To this end, several studies have shown that thermal therapy can enhance the therapeutic efficacy of immunotherapy when combined. For instance, a combinational protocol utilizing IL-2 or GM-CSF along with hyperthermia resulted in complete eradication of tumors in melanoma-bearing mice^71^. Finally, pyroptosis is another type of programmed cell death involving the activation of caspase-1^72^. This distinct pathway has protective effects against microbial infections for the host while a recent report revealed the bidirectional crosstalk between apoptosis and pyroptosis in innate immune cells^73^.

**Figure 8:**
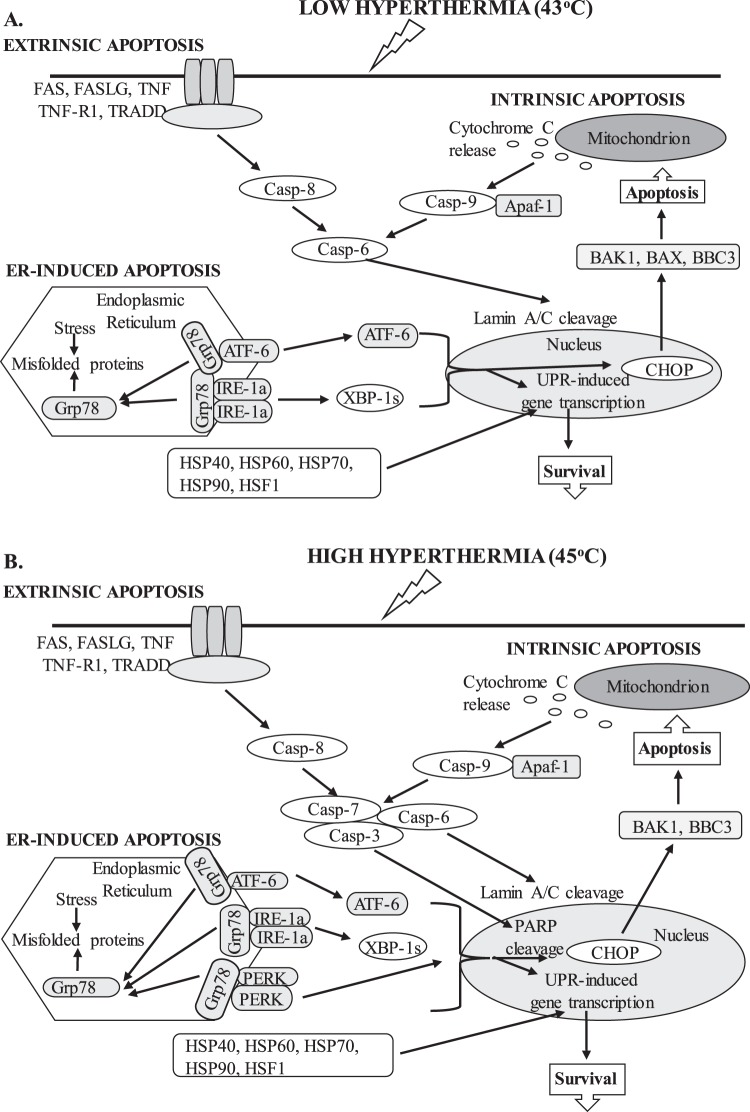
Schematic representation of proposed apoptotic induction in response to 43 °C (**A**) and 45 °C (**B**) hyperthermic exposures in human malignant melanoma (A375) cells.

Collectively, our data suggest that at higher temperatures (45 °C) cells could not adapt effectively and consequently increased cytotoxicity and apoptotic cell death were evident whereas at milder hyperthermic conditions (43 °C) the cells were more thermotolerant and thus able to regulate the apoptotic response in a more efficient manner. For instance, although initiator caspases -8 and -9 were activated in response to both 43 °C and 45 °C, induction of effector caspases appeared to differ between the two hyperthermic conditions in a manner where triggering of effector caspases-3, -7 and -6 occurred at 45 °C (Fig. 8B) whereas only caspase-6 was activated at 43 °C (Fig. 8A). This suggests that mild hyperthermia triggers the apoptotic response in a more regulated manner in contrast to more excessive hyperthermia which requires the participation of all the executioner caspase repertoire in order to sustain apoptotic cell death. Moreover, this study provides further insights in the involvement of ATF-6, IRE-1 and PERK in regulating the apoptotic activation in response to low and high hyperthermic conditions. More specifically, it was evident that only IRE-1a and ATF-6 pathways were induced at 43 °C (Fig. 8A) whereas all three of them were activated at 45 °C (Fig. 8B). Although both the IRE-1 and ATF6 pathways can up-regulate CHOP, PERK predominates through selective up-regulation of translation of ATF4 which, in turn, induces transcription of CHOP. Hence, it can be proposed that PERK signaling along with the subsequent induction of CHOP play a major role in regulating hyperthermia-induced apoptosis. Last but not least, hyperthermia exerted a significant role in potentiating the therapeutic effectiveness of a number of non-targeted and targeted drugs (when administered as adjuvant treatment protocols) thus high lightening its premise as a therapeutic approach in melanoma patients.

## Materials and Methods

### Cell lines

The human epidermoid carcinoma (A431) and malignant melanoma (A375) cell lines were purchased from Sigma-Aldrich (St. Louis, MO, USA). The human immortalized keratinocyte (HaCaT) cell line was a kind gift from Dr. Sharon Broby (Dermal Toxicology and Effects Group; Centre for Radiation, Chemical and Environmental Hazards; Public Health England, UK). All cell lines were maintained in Dulbeccos’s Modified Eagle Medium (DMEM), high glucose, supplemented with 10% fetal bovine serum, 2 mM L-glutamine and 1% pen/strep (100U/ml penicillin, 100 μg/ml streptomycin). Cells were cultured in a humidified atmosphere at 37 °C and 5% CO_2_. They were grown as monolayer cultures and sub-cultured when reaching 80–90% confluence. All cell lines were cultured for up to 20–25 passages before new vials were utilized. All cell culture media and reagents were purchased from Labtech International Ltd (East Sussex, UK) and cell culture plastic ware were obtained from Corning (NY, USA).

### Exposure to hyperthermia

Cells were exposed to a range of temperatures (37 °C–50 °C) for various time periods in a standard 5% CO_2_ incubator. Briefly, the appropriate number of cells was plated and incubated at 37 °C overnight. Next day, medium was changed prior to hyperthermic exposure and all plates were transferred into a 5% CO_2_ incubator set at 37–50 °C and exposed for various time periods. Then, plates were returned at a 37 °C incubator for additional incubation periods (post-exposure).

### Adjuvant hyperthermic exposures

Cells were seeded into 96-well plates and incubated at 37 °C, overnight. On the following day, plates were transferred into an incubator set at 43 °C (hyperthermia) or 37 °C (untreated) for 2 h. After the end of the exposure period, medium was aspirated and various concentrations of non-targeted (Dacarbazine, Temozolomide) and targeted (Dabrafenib, Vemurafenib) drugs were added over a time course of 24–72 h at 37 °C. Dacarbazine and Temozolomide were purchased from Abcam (Cambridge, UK) and Sigma-Aldrich (St. Louis, MO, USA) respectively while Dabrafenib and Vemurafenib were obtained from Selleckchem (Houston, TX, USA).

### Determination of cell viability and cytotoxicity

Cells were seeded in 96 well-plates with 100 μl medium and incubated overnight before hyperthermic exposures. Prior to exposures, the medium was refreshed and cells were exposed to various hyperthermic conditions at the end of which they were returned back to 37 °C. Cell viability levels were determined immediately after exposures as well as at 24–72 h post-exposure by utilizing the Celltiter-Blue Assay (Promega, UK) according to the manufacturer’s protocol. The assay uses the indicator dye (resazurin) which is converted to a highly fluorescent product (resorufin) by metabolically active cells. Non-viable cells lose their metabolic capacity; thus, they are not able to reduce resazurin into the fluorescent product and consequently cannot generate a fluorescent signal. Briefly, 20 μl of Celltiter-Blue reagent was added into each well of α 96-well plate and mixed by gentle shaking. The plates were incubated at 37 °C for 2 h and then the samples were transferred into the wells of a black opaque plate. Fluorescence was monitored at 400 Exc/505 Emm (nm) by using a SpectraMax M5 multimode plate reader (Molecular Devices, LLC, Sunnyvale, USA). Cell viability was expressed as percentage of control (37 °C) cells. Five replicates (n = 5) of each experimental condition were used under each experiment.

Determination of relative levels of dead cells was made based on the CytoTox-Fluor cytotoxicity assay (Promega, UK) according to the manufacturer’s protocol. The assay involves a fluorogenic peptide substrate (bis-alanyl-alanyl-phenylalanyn-rhodamine 110; bis-AAF-R110) which can measure the activity levels of a specific protease released from dead cells which have lost membrane integrity. This particular peptide substrate cannot produce a signal in viable cells as it cannot cross their cell membrane. Briefly, cells were plated in 96 well-plates, exposed to hyperthermic conditions and then 100 μl of the assay reagent was added into each well (at indicated time points) mixed by orbital shaking and incubated at 37 °C for 2 h. Then, samples were transferred into the wells of a black opaque plate and fluorescence was monitored at 400 Exc/505 Emm (nm) by using a SpectraMax M5 multimode plate reader. The generation of fluorescent product is proportional to the protease activity of the marker associated with cytotoxicity so that higher fluorescence values represent increased levels of dead cells. Five replicates (n = 5) of each condition were used in each experiment.

In another approach, the trypan blue staining protocol was utilized in order to determine levels of viable and dead cells within the same sample. Briefly, cells were plated in 100 mm^3^ dishes (incubated overnight at 37 °C) and after exposure to hyperthermia they were trypsinized and collected. A sample of each cell suspension was mixed with the trypan blue stain and cells were counted under the microscope. Overall, cells were categorized into being either viable (unstained) or dead (stained) while the total cell suspension number was calculated. Three replicates (n = 3) of each experimental condition were used under each experiment.

### RNA extraction and determination of apoptotic gene profiling by RT-PCR-based microarrays

To examine differential apoptotic gene expression in response to hyperthermia, A375 cells were plated in 100 mm cell culture dishes, cultured overnight and exposed to 43 °C and 45 °C or 37 °C for 2 h. Cells were then returned to 37 °C for an additional 24 h incubation period after which they were collected via trypsinization. Total RNA was extracted using the TRIzol reagent according to the manufacturer’s protocol (Invitrogen). RNA quality and concentration were assessed by agarose gel electrophoresis and spectrophotometric analysis. Complimentary DNA was synthesized by using the SuperScript VILO cDNA synthesis kit (Invitrogen, Waltham, MA, USA) according to the manufacturer’s protocol. qPCR was carried out by utilizing the TaqMan Array Human Apoptosis 96-well plates (Applied Biosystems, Carlsbad, CA, USA). TaqMan Universal master mix (2x) was mixed with the equal amount of diluted cDNA (5–50 ng per well) in RNAase free water and 10 μl of the mixture were added into each well of the 96-well plate. RT-PCR was performed on a StepOne Plus RT-PCR system (Applied Biosystems, Carlsbad, CA, USA). Gene expression data were analyzed by the ΔΔCt method and differences observed were expressed as fold change in gene expression by using the DataAssist v3.01 software.

### Determination of protein expression by western blotting

Samples were stored as cell pellets at −20 °C following trypsinization and PBS washes. Cell pellets were suspended in the appropriate amount of lysis buffer (10 mM HEPES, pH 7.9; 10 mM KCl; 0.1 mM EDTA; 1.5 mM MgCl_2_; 0.2% NP-40) supplemented with a cocktail of protease inhibitor tablets (Thermo Fisher, Waltham, MA, USA), and were left on ice while periodically being vortexed for 15 min. Then, they were sonicated at 30% amplitude for 3 cycles of 15 s each (with 30 s intervals) on ice. Cell lysates were centrifuged at 14,000 × g for 15 min at 4 °C and protein content was determined by utilizing the Pierce BCA protein assay kit according to the manufacturer’s protocol. Fifty μg of proteins were separated by using SDS-polyacrylamide gels of different gradient (8–20%) according to the molecular weight of the protein of interest. Separated proteins were then transferred electrophoretically onto either 0.2 and/or 0.45μm PVDF membranes (depending on protein’s molecular weight) (Thermo Scientific, Waltham, MA, USA) by wet transfer in 1x transfer buffer at predetermined running conditions. The blots were blocked with 5% (w/v) non-fat milk powder in TBST buffer, for 1 h at RT, under gentle agitation. Then, the blots were incubated with specific primary antibodies, overnight at 4 °C, under gentle agitation. On the following day, the membranes were washed in TBST buffer for 10 min, three times, and then were incubated with an appropriate secondary antibody, for 1 h at RT, under agitation. Blots were incubated with SuperSignal West Pico Chemiluminescent Substrate (Thermo Scientific, Waltham, MA, USA) according to the manufacturer’s protocol before being imaged by using a ChemiDoc XRS^+^ system (Bio-Rad, Perth, UK). All antibodies were purchased from Cell Signaling Technology (Hertfordshire, UK), apart from β-tubulin which was from Sigma-Aldrich (St. Louis, MO, USA).

### Data analysis

Experimental conditions for all sets of experiments were expressed as mean values ± SEM and comparisons were made between control and treatment groups. Calculations were performed by using the Microsoft Office Excel 2016 software. Means were compared by one-way analysis of variance (one-way ANOVA) with Tukey’s test for multiple comparisons. SPSS v.22 or PRISM v5.01 software were used for statistical tests. A value of p < 0.05 was considered statistically significant.

## Electronic supplementary material

Supplementary Information
