# Supplementary material for: Hyperthermia induces therapeutic effectiveness and potentiates adjuvant therapy with non-targeted and targeted drugs in an in vitro model of human malignant melanoma
Source: Sci Rep. 2018 Jul 16;8:10724. doi: 10.1038/s41598-018-29018-0 (PMC6048057; doi:10.1038/s41598-018-29018-0)
Supplement: Supplementary file 1 — Supplementary Information [file 41598_2018_29018_MOESM1_ESM.pdf]

Hyperthermia induces therapeutic effectiveness and potentiates adjuvant therapy with non-targeted and targeted drugs in an *in vitro* model of human malignant melanoma

Mantso T[1,2], Vasileiadis S[3], Anestopoulos I[4], Voulgaridou GP[4], Lampri E[5], Botaitis S[6], Kontomanolis EN[3], Simopoulos C[6], Gousetis G[7], Franco R[8,9], Chlichlia K[4], Pappa A[4], Panayiotidis MI[2,#]

[1]School of Life Sciences, Heriot Watt University, Edinburgh, Scotland, UK; [2]Department of Applied Sciences, Northumbria University, Newcastle Upon Tyne, UK; [3]Department of Obstetrics & Gynecology, Democritus University of Thrace, Alexandroupolis, Greece; [4]Department of Molecular Biology & Genetics, Democritus University of Thrace, Alexandroupolis, Greece; [5]Department of Pathology, University of Ioannina, Ioannina, Greece; [6]Second Department of Surgery, Democritus University of Thrace, Alexandroupolis, Greece; [7]School of Engineering & Physical Sciences, Heriot Watt University, Edinburgh, Scotland, UK; [8]Redox Biology Centre and [9]School of Veterinary Medicine & Biomedical Sciences, University of Nebraska, Lincoln, USA

[#]Corresponding author:

Prof. Mihalis I. Panayiotidis, PhD

Department of Applied Sciences, Faculty oh Health and Life Sciences, Northumbria University, Ellison Building A516, Newcastle Upon Tyne, NE1 8ST; United Kingdom

Tel: +44(0)1912274503

E-mail: m.panagiotidis@northumbria.ac.uk

Supplementary Figures

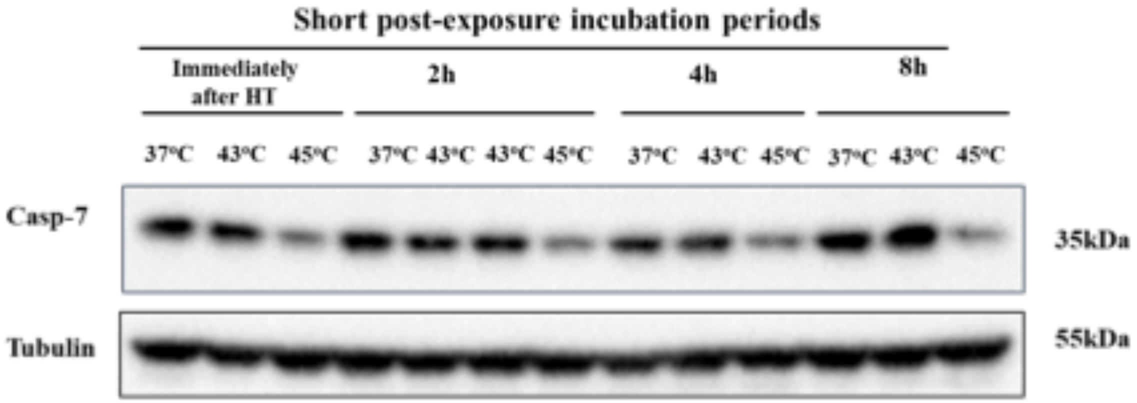

Figure 1S. Hyperthermia-induced apoptosis in a human malignant melanoma (A375) cell line. The effect of hyperthermia on protein content of caspase-7. Full-length blot for short-exposure incubation periods is provided due to double loading for the 2h time point following exposure at 43°C. Cells were grown overnight at 37°C followed by exposure to hyperthermia, for 2h, and then transferred back to 37°C for the indicated post-exposure incubation times (2-72h). Cell lysates were prepared and subjected to western blotting. Control cells were kept at 37°C. β-tubulin was used as loading control. Data shown is representative of at least two independent experiments.

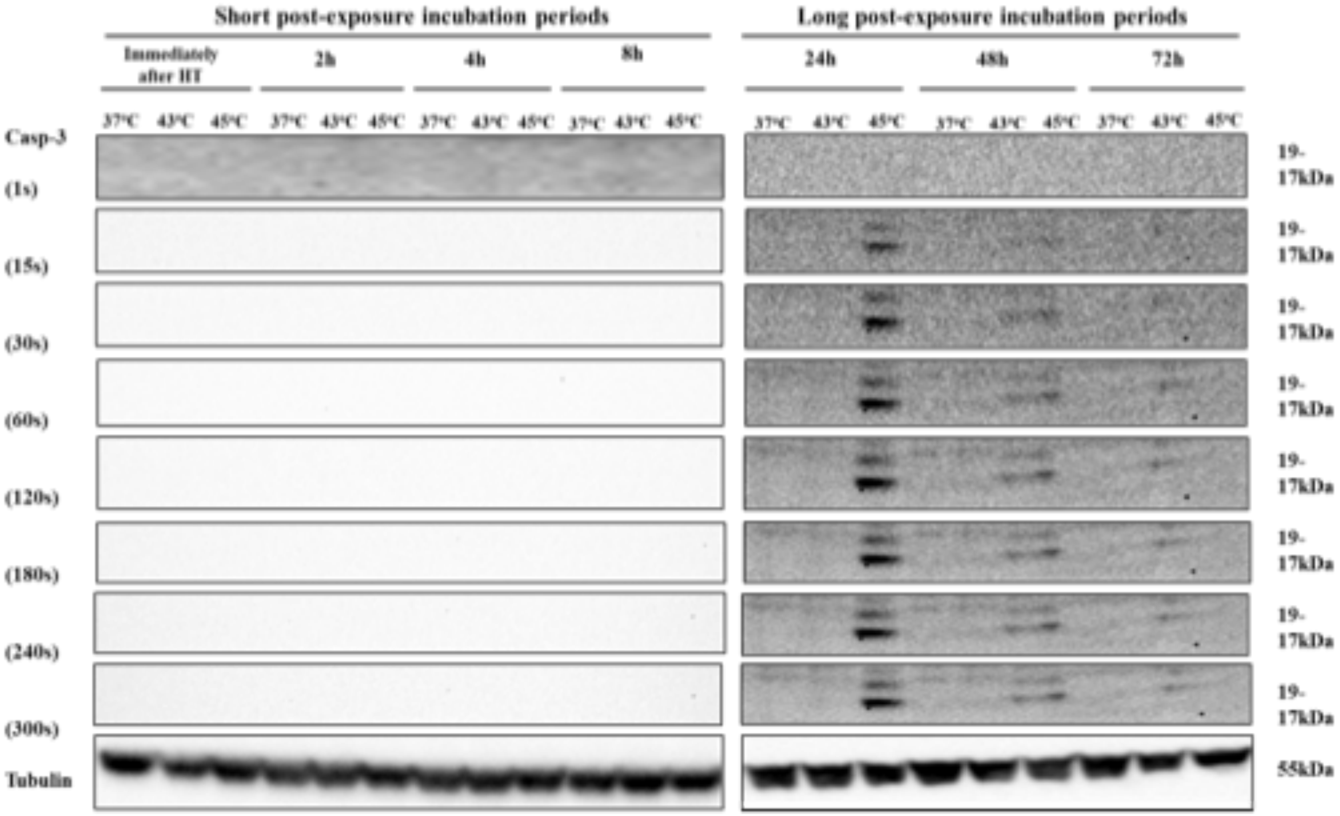

Figure 2S. Hyperthermia-induced apoptosis in a human malignant melanoma (A375) cell line. The effect of hyperthermia on protein content of caspase-3 (19-17kDa fragment). Multiple imaging exposures (1. 15, 30, 60, 120, 180, 240 and 300s) are provided. Cells were grown overnight at 37°C followed by exposure to hyperthermia, for 2h, and then transferred back to 37°C for the indicated post-exposure incubation times (2-72h). Cell lysates were prepared and subjected to western blotting. Control cells were kept at 37°C. β-tubulin was used as loading control. Data shown is representative of at least two independent experiments.

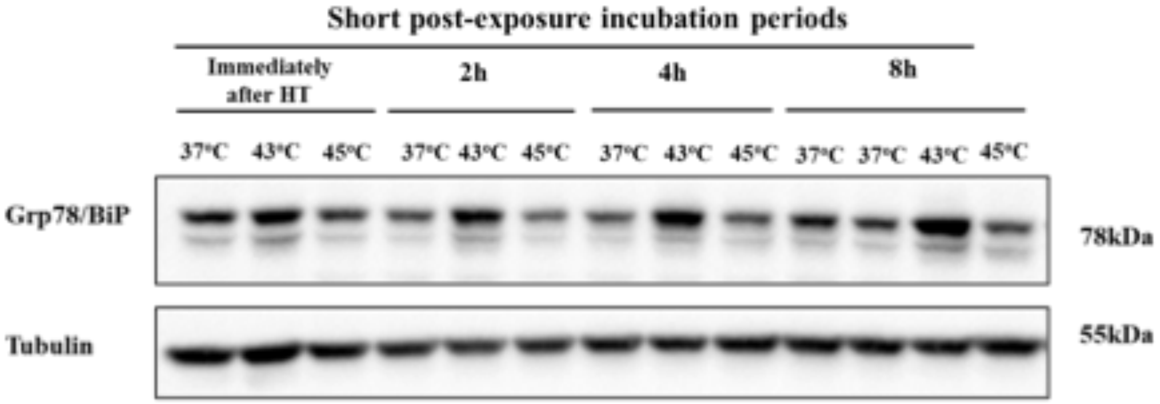

Figure 3S. Hyperthermia induces ER stress response in human malignant melanoma (A375) cells. The effect of hyperthermia on protein content of Grp78/BiP. Full-length blot for short post-exposure incubation periods is provided due to double loading for the 8h time point following exposure at 37°C. Cells were grown overnight at 37°C followed by exposure to hyperthermia, for 2h, and then transferred back to 37°C for the indicated post-exposure incubation times (2-72h). Cell lysates were prepared and subjected to western blotting. Control cells were kept at 37°C. β-tubulin was used as loading control. Data shown is representative of at least two independent experiments.
